# The relationship of polluted air and drinking water sources with the prevalence of systemic lupus erythematosus: a provincial population-based study

**DOI:** 10.1038/s41598-021-98111-8

**Published:** 2021-09-20

**Authors:** Jiaqi Chen, Wenqiang Qu, Li Sun, Jiansheng Chen, Wei Kong, Fan Wang, Wenyou Pan, Lin Liu, Min Wu, Fuwan Ding, Huaixia Hu, Xiang Ding, Hua Wei, Yaohong Zou, Xian Qian, Meimei Wang, Jian Wu, Juan Tao, Jun Tan, Zhanyun Da, Miaojia Zhang, Jing Li, Jun Liang, Xuebing Feng, Linyu Geng, Lingyun Sun

**Affiliations:** 1 School of Computer and Information, Hohai University, Nanjing, China; 2 School of the Environment, Nanjing University, Nanjing, China; 3 School of Earth Science and Engineering, Hohai University, Nanjing, China; 4 Department of Rheumatology and Immunology, The Affiliated Drum Tower Hospital of Nanjing University Medical School, 321 Zhongshan Road, Nanjing, 210008 China; 5 Department of Rheumatology, Huai’an First People’s Hospital, Huai’an, China; 6 Department of Rheumatology, Xuzhou Central Hospital, Xuzhou, China; 7 Department of Rheumatology, The Third Affiliated Hospital of Soochow University, Changzhou, China; 8 Department of Endocrinology, Yancheng Third People’s Hospital, Yancheng, China; 9 Department of Rheumatology, The Second People’s Hospital of Lianyungang, Lianyungang, China; 10 Department of Rheumatology, The First People’s Hospital of Lianyungang, Lianyungang, China; 11 Department of Rheumatology, Northern Jiangsu People’s Hospital, Yangzhou, China; 12 Department of Rheumatology, Wuxi People’s Hospital, Wuxi, China; 13 Department of Rheumatology, Jiangsu Province Hospital of Traditional Chinese Medicine, Nanjing, China; 14 Department of Rheumatology, Southeast University Zhongda Hospital, Nanjing, China; 15 Department of Rheumatology, The First Affiliated Hospital of Soochow University, Suzhou, China; 16 Department of Rheumatology, Wuxi TCM Hospital, Wuxi, China; 17 Department of Rheumatology, Zhenjiang First People’s Hospital, Zhenjiang, China; 18 Department of Rheumatology, Affiliated Hospital of Nantong University, Nantong, China; 19 Department of Rheumatology, Jiangsu Province Hospital, Nanjing, China; 20 Department of Rheumatology, Affiliated Hospital of Jiangsu University, Zhenjiang, China

**Keywords:** Environmental sciences, Hydrology, Diseases, Medical research, Pathogenesis, Rheumatology

## Abstract

Environmental exposures interact with genetic factors has been thought to influence susceptibility of systemic lupus erythematosus (SLE) development. To evaluate the effects of environmental exposures on SLE, we conducted a population-based cohort study across Jiangsu Province, China, to examine the associations between the living environment including air and water pollution, population density, economic income level, etc. and the prevalence and mortality of hospitalized SLE (h-SLE) patients. A total of 2231 h-SLE patients were retrieved from a longitudinal SLE database collected by the Jiangsu Lupus Collaborative Group from 1999 to 2009. The results showed that: It existed regional differences on the prevalence of h-SLE patients in 96 administrative districts; The distribution of NO_2_ air concentration monitored by atmospheric remote sensors showed that three of the ultra-high-prevalence districts were located in the concentrated chemical industry emission area; h-SLE patient prevalence was positively correlated with the excessive levels of nitrogen in drinking water; The positive ratio of pericarditis and proteinuria was positively correlated with the prevalence of h-SLE patients and pollution not only induced a high h-SLE patient prevalence but also a higher mortality rate, which might be attributed to NOx pollution in the air and drinking water. In summary, our data suggested that NOx in air and drinking water may be one of the important predispositions of SLE, especially for patients with renal involvement.

## Introduction

Systemic lupus erythematosus (SLE) is a multi-systemic chronic autoimmune disease with multifactorial etiology^1^. Evidence support that multiple factors contribute to SLE pathogenesis and clinical manifestations, including genetic susceptibility, environmental effects, and disturbances in both innate and adaptive immunity manifest^2–6^.

The possibility that virus may trigger SLE has been considered during the past 40 years^7–9^. Evidence for an environmental component in SLE arises from observations that the majority of lupus cases are idiopathic, drugs such as procainamide, hydralazine and other factors including smoking as well as UV light trigger lupus-like autoimmunity^10–12^, and the incomplete concordance between genetically identical twins^13^. Recently, environmental pollution has been threatening human health. The increased air pollutant concentration levels are positively correlated with the risk of SLE, NO2, SO2, CO, O3 and so on in the air are all considered to be potential risk factors for systemic inflammation in patients with SLE^14^. A nationwide population-based case–control study from Taiwan, China, support that the development of SLE was positively associated with NO_2_ exposure, but negatively with CO exposure^15^. Fernandes et al. explored the relationship between exposure to air pollutants and disease activities in 22 children with SLE (c-SLE), and found that exposure under PM10, NO_2_ and CO for about 2 weeks was a risk factor for c-SLE activity (SLEDAI-2K > 8), and moving average of the PM10 increased to 13.4 μg/m^3^ could increase the risk of SLEDAI-2K > 8 by 34%^16^. Alves et al. conducted a longitudinal panel study of 108 repeated measurements of 9 children with lupus for one year, and found that NO_2_ level was associated with decreased serum C3 levels^17^.

Pollution from drinking water sources has been proven to be the cause of SLE. Ingestion of NO_3_^-^ can cause methemoglobinemia^18^, further, virus, bacteria, heavy metals, pesticide residues such as agricultural pesticides and antibiotics, industrial wastewater, agricultural non-point source pollution, domestic sewage and other pollutants entering the drinking water may increase the risk of SLE^19,20^. The comparative experimental analysis results of single chemicals in drinking water and the incidence of SLE all show a certain correlation^21^. The environment may be associated with epigenetic changes caused by exposure, increased oxidative stress, systemic inflammation and upregulation of inflammatory cytokines, and hormonal effects. Environmental agents can alter T cell gene expression through effects on DNA methylation, resulting in autoreactive T cells that promote autoimmunity^22^. Further, ERK pathway signaling is an important regulator of DNA methyltransferase 1 (DNMT1) that is decreased in hydralazine-treated T cells and in T cells from patients with idiopathic lupus^23^. Therefore, environmental agents that inhibit ERK signaling, its upstream regulator protein kinase C delta (PKC-d), or other conditions such as diet and aging, that impact DNMT1 activity may increase methylation-sensitive gene expression through epigenetic mechanisms to cause a lupus-like disease in genetically predisposed individuals^24,25^. All of these data support that environmental pollutants have proinflammatory effects and are therefore contributing to the generation of inflammation and autoimmunity.

However, the correlation between air and water pollutions and SLE development is still largely unknown, because it is difficult to rule out the living environment interference factors of each patient in the experimental group, and rare multi-center, large sample, systematic retrospective analysis was reported, which limit the effective and targeted solutions for the prevention of SLE prevalence induced by air and water pollutions. Here we investigated the living environment of 2231 hospitalized SLE (h-SLE) patients distributed in 96 administrative districts of 13 cities in Jiangsu Province, China, from 1999 to 2009, and analyzed the dataset for interrelations and mutual spatial occurrence to examine the relationship between the living environment including air and water pollution, population density, economic income level, etc. and the prevalence of h-SLE patients. Our data showed NOx in air and drinking water may be one of the important predispositions of SLE, especially for patients with renal involvement, while the economic and living standards had no effect on h-SLE patient prevalence distribution.

## Materials and methods

### Study population

A total of 2231 individuals were retrieved from a longitudinal SLE database collected by the Jiangsu Lupus Collaborative Group from 1999 to 2009. The distribution of the year of admission and diagnosis of 2231 patients, who were all hospitalized for the first time, was shown in Supplementary Fig. S1A,B, and more than 70% patients were initially diagnosed with SLE (Supplementary Fig. S1C), 44% patients were severe SLE (SLEDAI score > 14) patients (Supplementary Table S1). The demographic and clinical characteristics data of h-SLE patients was presented in Table 1 and Supplementary Table S2, and SLEDAI distribution of these patients was shown in Supplementary Fig. S1D.

**Table 1:** Demographic and clinical characteristics of h-SLE patients.

Variable	Total (n = 2231)	South of Yangtze (n = 1186)	North of Yangtze (n = 1045)	P value
Age, years (Mean ± SD)	33.5 ± 12.6	34.2 ± 12.7	32.8 ± 12.4	0.0252
Female (%)	2062 (92.4)	1098 (92.6)	964 (92.2)	0.8298
Male (%)	169 (7.6)	88 (7.4)	81 (7.8)	0.8298
SLEDAI on admission	14.6 ± 7.9	14.3 ± 7.8	14.9 ± 8.0	0.1366
**Organ involvements**				
Mucocutaneous	1461 (65.5)	703 (61.6)	758 (72.5)	0.0000
Neuropsychiatric	138 (6.2)	56 (4.7)	82 (7.8)	0.0030
Musculoskeletal	1242 (55.7)	614 (51.8)	628 (60.1)	0.0001
Cardiopulmonary	473 (21.2)	271 (22.8)	202 (19.3)	0.0480
Gatrointestinal	108 (4.8)	52 (4.4)	56 (5.4)	0.3315
Ocular	9 (0.4)	6 (0.5)	3 (0.3)	0.6319
Renal	1146 (51.4)	633 (53.4)	513 (49.1)	0.0481
Haematological	1055 (47.3)	593 (50.0)	462 (44.2)	0.0071
**Serology**				
Anti-dsDNA positive	1221 (54.7)	661 (55.7)	560 (53.6)	0.3305
Anti-Sm positive	659 (29.5)	342 (28.8)	317 (30.3)	0.4668
Anti-cardiolipin positive	228 (10.7)	96 (8.1)	132 (12.6)	0.0005
RF positive	425 (19.0)	250 (21.1)	175 (16.7)	0.0109

Statistical analysis was performed with the Mann–Whitney U-test and the χ^2^ test.

*SLEDAI* SLE disease activity index.

### Measurement of NO_2_ concentration in Jiangsu

The ozone monitoring instrument (OMI) equipped with the AURA satellite was launched in 2004 to monitor NO_2_ concentration. The OMI detector has a spatial resolution of 13 km × 24 km, and could cover the world every day. We selected the average data of NO_2_ tropospheric column concentration in August 2005. Due to the complexity of the spatial resolution of the original raster data, we first converted the original raster data into points vector data in ARCGIS10.2, and then used Kriging space interpolation method for point vector data to obtain the spatial distribution map of NO_2_ concentration in Jiangsu Province, at the same time, the patient’s geographic coordinate information was used to project the patient onto the concentration distribution map, and then regional statistical analysis was used to calculate the NO_2_ concentration in each administrative district.

### Measurement of Night light in Jiangsu

The first generation of luminous data is derived from the Operational Linescan System (OLS) carried by the Defense Meteorological Satellite Program (DMSP) in the last century, which has the world's longest time series data stock (1992–2013). The luminous radiation signal on the DMSP/OLS image is discretized into digital number values (DN value, range 0–63), and the area covered by a high DN value usually corresponds to an area with a higher level of human activity and economic development. We selected the 2003 DMSP/OLS image, and through related processing including cropping, denoising, got luminous distribution map in Jiangsu Province, and used the patient's geographic coordinate information to project the patients onto the concentration distribution map, and then used regional statistical analysis to calculate night light value in each administrative district.

### Measurement of Water chemistry type in Jiangbei

Shi et al. investigated and discussed the characteristics and evolution of groundwater quality in the northern plains of Jiangsu Province on the basis of field data including 597 data collected in the 1980s and 1346 samples analyzed in the 2000s. We referred to the cation distribution map of the water chemical phase of the diving aquifer in the 2000s in the paper published by him, draw the distribution map of the cation water chemistry type in northern Jiangsu, and estimated the h-SLE patient prevalence of each type of water chemistry type based on the proportion of the element coverage area, and projected the patients to the water chemistry type distribution map.

### Statistical analysis

Data statistics adopt district, classification and level statistics, and datasets were analyzed for interrelations and mutual spatial occurrence using statistical approaches including correlation analysis and significant difference analysis.

SPSS software version 22.0, GraphPad Prism version 8.0, Microsoft Excel version 2019 and Python version 3.7 were used for statistical analyses. Continuous data were expressed as means and standard deviations, whereas Categorical data were presented as a percentage. Non-normally distributed data were analyzed using the Mann–Whitney U-test. Categorical data were compared by means of the χ^2^ test. The prevalence and mortality of h-SLE patients conformed to the normal distribution after BOX-COX conversion and Shapiro–Wilk Test, and then divided the 96 administrative districts into 5 groups according to value of Night light, NO_2_ concentration, and population density, and assuming that each group was independent of each other, a multi-batch box-plot was used to compare and analyze the distribution shape, dispersion, and central tendency of different groups and different types of data. Correlations between the Night light value, NO_2_ concentration, and h-SLE patient prevalence were established using pearson correlation analysis and univariate regression analysis. Intergroup differences in Night light value, population density, NO_2_ concentration, water chemistry type was analyzed by one-way analysis of variance. The medical indicators were counted separately according to different h-SLE patient prevalence level and various indicators in districts of Jiangnan and Jiangbei were analyzed. P values of less than 0.05 were considered to be statistically significant.

### Ethical considerations

The study complied with the ethical principles of the Declaration of Helsinki. Written informed consent was obtained from all patients. This study has been approved by the Ethics Committee of the Affiliated Drum Tower Hospital of Nanjing University Medical School.

## Results

### Environmental exposures might be triggering the development of h-SLE

**Figure 1:**
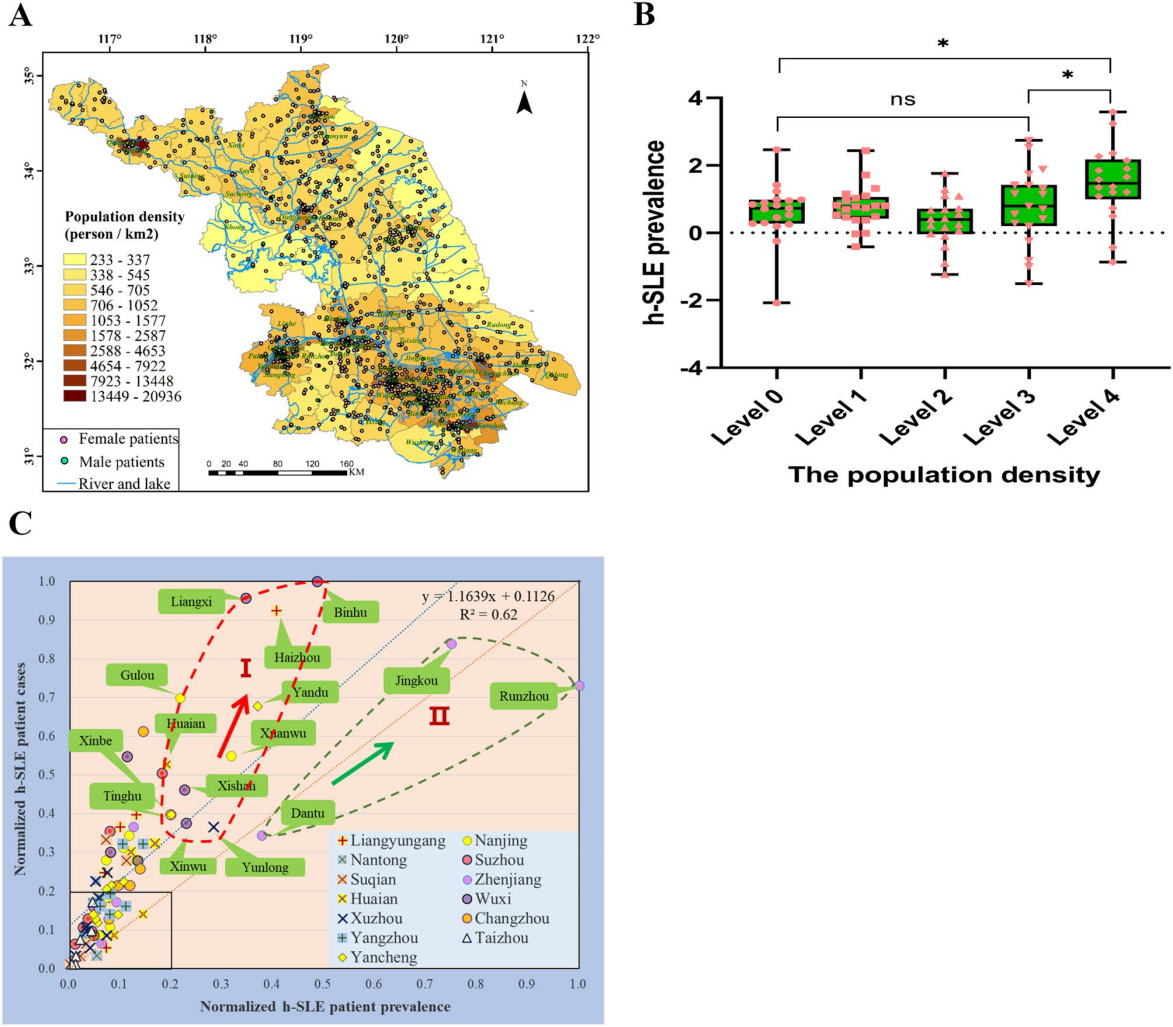
Environmental exposures might be triggering the development of h-SLE. (**A**) Population density and distribution of h-SLE patients in Jiangsu Province (prepared by WQ in ArcMap 10.2, https://www.esri.com/zh-cn/arcgis/products/arcgis-pro/resources). (**B**) Significant difference analysis of the h-SLE patient prevalence of population density (person/km^2^) group. Level 0:230–560; Level 1: 560–700; Level 2: 700–1000; Level 3: 1000–2000; Level 4:2000–20,100. (**C**) Normalized relationship between the prevalence of h-SLE patients and the number of patient cases in Jiangsu Province, including 10 in the south of the Yangtze River and 5 in the north of the Yangtze River. The prevalence of h-SLE patients conformed to the normal distribution after BOX-COX conversion (λ = 0.053) and Shapiro–Wilk Test (P value: 0.186 > 0.05). Statistical analysis was performed with one-way ANOVA. *P < 0.05, *ns* not significant.

The group difference analysis in Table1 showed that the two groups of patients in Jiangnan and Jiangbei did not show great differences in the demographic and clinical characteristics. To evaluate the population density of each administrative district and the distribution of h-SLE patients, we analyzed the density of 2231 individuals in 96 administrative districts of Jiangsu Province (Fig. 1A) and significant difference analysis of the h-SLE patient prevalence of population density (person/km^2^) group indicated that there was no significant difference in h-SLE patient prevalence among different groups (Fig. 1B). The highest prevalence of h-SLE patients appeared in Runzhou, Zhenjiang (26.6 cases per 100,000 people), and the lowest prevalence was in Sihong, Suqian (0.11 cases per 100,000 people), with a ratio of up to 240. Significant regional difference in patient prevalence suggested that environmental exposures might be crucial triggers for lupus. To reveal the relationships of environmental exposures and h-SLE incidence, we conducted a normalized analysis between the cases and the h-SLE patient prevalence in 96 administrative districts of Jiangsu Province (R^2^ = 0.62, Fig. 1C). 45 of the 96 districts fell outside the 0.2 × 0.2 area, supporting that the onset of lupus may be triggered by exposure to environmental factors. The 15 high-prevalence districts (> 5 cases per 100,000 people) were distributed in two different Zones I and II, among which, 3 ultra-high-prevalence districts (> 10 cases per 100,000 people) distributed in Zone II, Runzhou, Jingkou and Dantu, are all located in Zhenjiang, the south bank of the Yangtze River, where a large number of chemical enterprises are gathered, indicating that the exhaust emission of centralized chemical plants might participate in the development of SLE.

### Relationship of the concentrated discharge of exhaust gas with the prevalence of h-SLE patients

**Figure 2:**
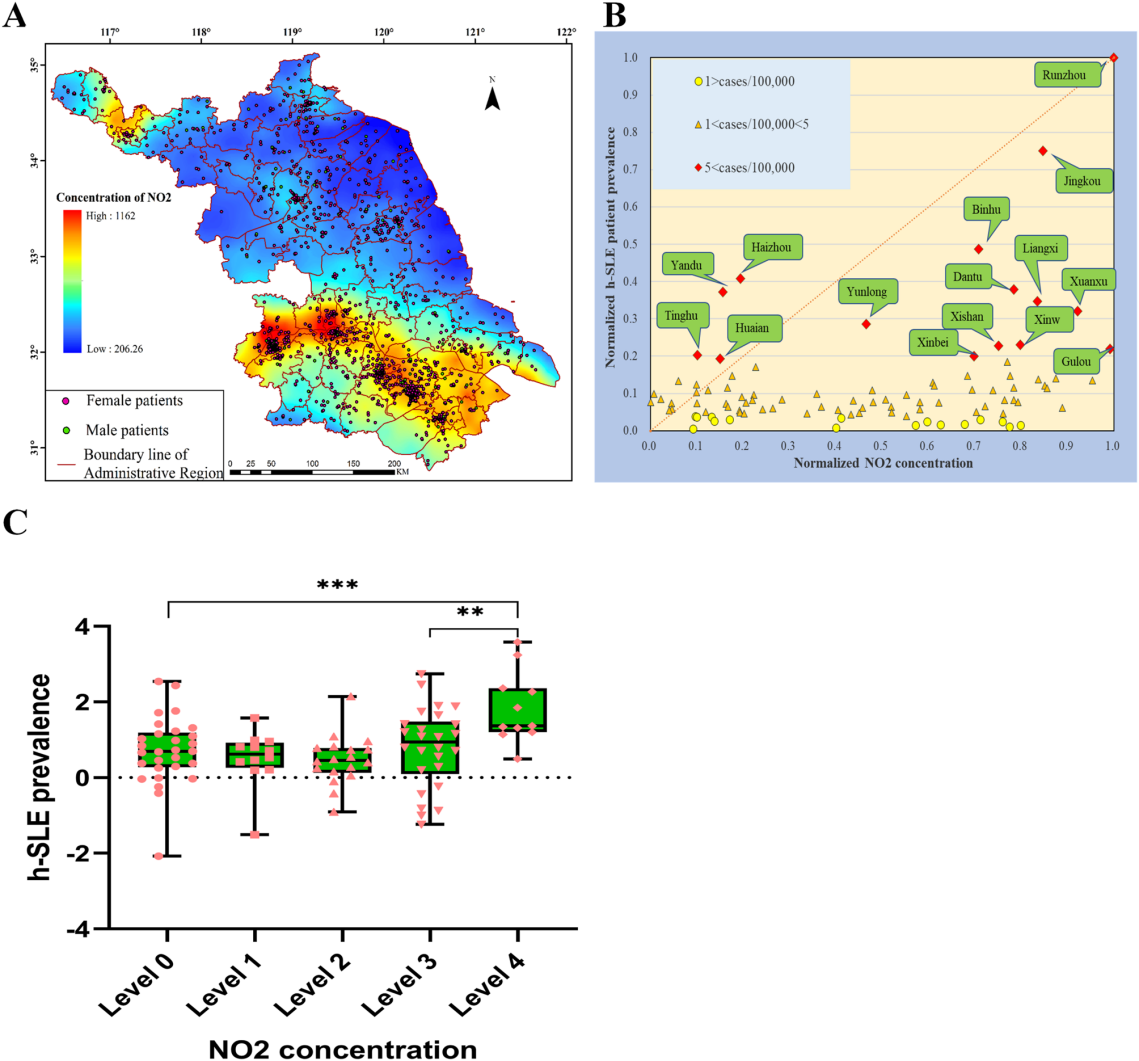
Association between the concentrated discharge of exhaust gas and the development of h-SLE. (**A**) NO_2_ concentration distribution in the air of Jiangsu Province in August 2005 (prepared by WQ in ArcMap 10.2, https://www.esri.com/zh-cn/arcgis/products/arcgis-pro/resources). (**B**) Normalized graph of h-SLE patient prevalence and air NO_2_ concentration in August 2005 in Jiangsu Province from 1999 to 2009. (**C**) Significant difference analysis of the prevalence of h-SLE patients in NO_2_ concentration group. Level 0:250–420; Level 1: 420–585; Level 2: 585–750; Level 3: 750–910; Level 4:910–1075. The prevalence of h-SLE patients conformed to the normal distribution after BOX-COX conversion (λ = 0.053) and Shapiro–Wilk Test (P value: 0.186 > 0.05). Statistical analysis was performed with one-way ANOVA. **P < 0.01, ***P < 0.001.

Mostly, air and water pollution in Jiangsu Province were caused by exhaust emission from chemical enterprises. To assess the associations between air pollution and SLE, we analyzed the NO_2_ concentration distribution in Jiangsu Province, an indicator of air pollution, using OMI satellite atmospheric remote sensing data (Fig. 2A). It existed high h-SLE patient prevalence in areas with high NO_2_ concentration. The emission of NO_2_ in the 3 ultra-high-prevalence districts, Runzhou, Jingkou and Dantu, with only 23.2% of Zhenjiang area, accounting for 87.5% of the total city pollution emission. Among the high-prevalence districts in Nanjing, Xuanwu District was located in the industrial zone, and the concentrations of air pollutants including PM10, SO_2_ and NO_2_ were the highest; The annual average concentration of NO_2_ in Xuanwu and Gulou District was higher than the national environmental standard (0.04 mg/m^3^), and the maximum concentration of NO_2_ was 0.35 mg/m^3^ in Gulou, where mainly came from vehicle exhaust emission. Together, our data supported that the development of SLE was partially attributed to the concentrated discharge of exhaust gas from chemical plants or vehicle exhaust emission.

To further investigate the role of environmental exposures in the development of SLE, we made a normalized relationship chart between the NO_2_ concentration in the air and the h-SLE patient prevalence (Fig. 2B). Among the 15 high-prevalence districts of h-SLE patients, the air pollution in Jiangbei was lighter, for example, the NO_2_ normalization coefficients of Haizhou in Lianyungang, Yandu in Yancheng, and Huaian in Huai'an were all less than 0.2, and the NO_2_ normalization coefficient of Yunlong in Xuzhou was 0.47. In the low-prevalence districts with prevalence < 1, the normalized NO_2_ concentration in the air was < 0.8, with 6 points falling between 0.6 and 0.8, and significant difference analysis of the h-SLE patient prevalence of NO_2_ concentration group indicated that there was a threshold for air pollution-induced SLE disease (Fig. 2C), when the degree of air pollution was less than the threshold, the probability of induced SLE disease would be greatly reduced, and for areas with low air pollution, there should be other causes of SLE.

### Relations of the concentrated discharge of exhaust water and the prevalence of h-SLE patients

Considering the sources of drinking water might be from polluted surface water or unpolluted groundwater, to evaluate the role of water pollution in h-SLE patient prevalence, we first analyzed the sources of drinking water in h-SLE patient high-prevalence districts (Supplementary Table S3), and found 5 out of 15 in the north of Yangtze River: Haizhou, Yandu, Tinghu, Huaian and Yunlong, were all from polluted surface water. For the other 4 high-prevalence districts in Wuxi City, the source of drinking water has been unified into Tai Lake since the 1970s, the nitrogen and phosphorus pollution in Tai Lake were serious because of the increasingly eutrophication, and the cross-sectional proportions of Class V and inferior Class V water quality were 15% and 85%, respectively, according to the China Hydrological Yearbook, from 1999 to 2009. However, for Yixing and Jiangyin in Wuxi, where the sources of drinking water were from Hengshan Reservoir and Yangtze River, respectively, the patient prevalence (2.2 cases per 100,000 people & 3.1 cases per 100,000 people) was much lower than that of Wuxi City from Tai Lake, suggesting that water pollution might play crucial role in the prevalence of SLE.

**Figure 3:**
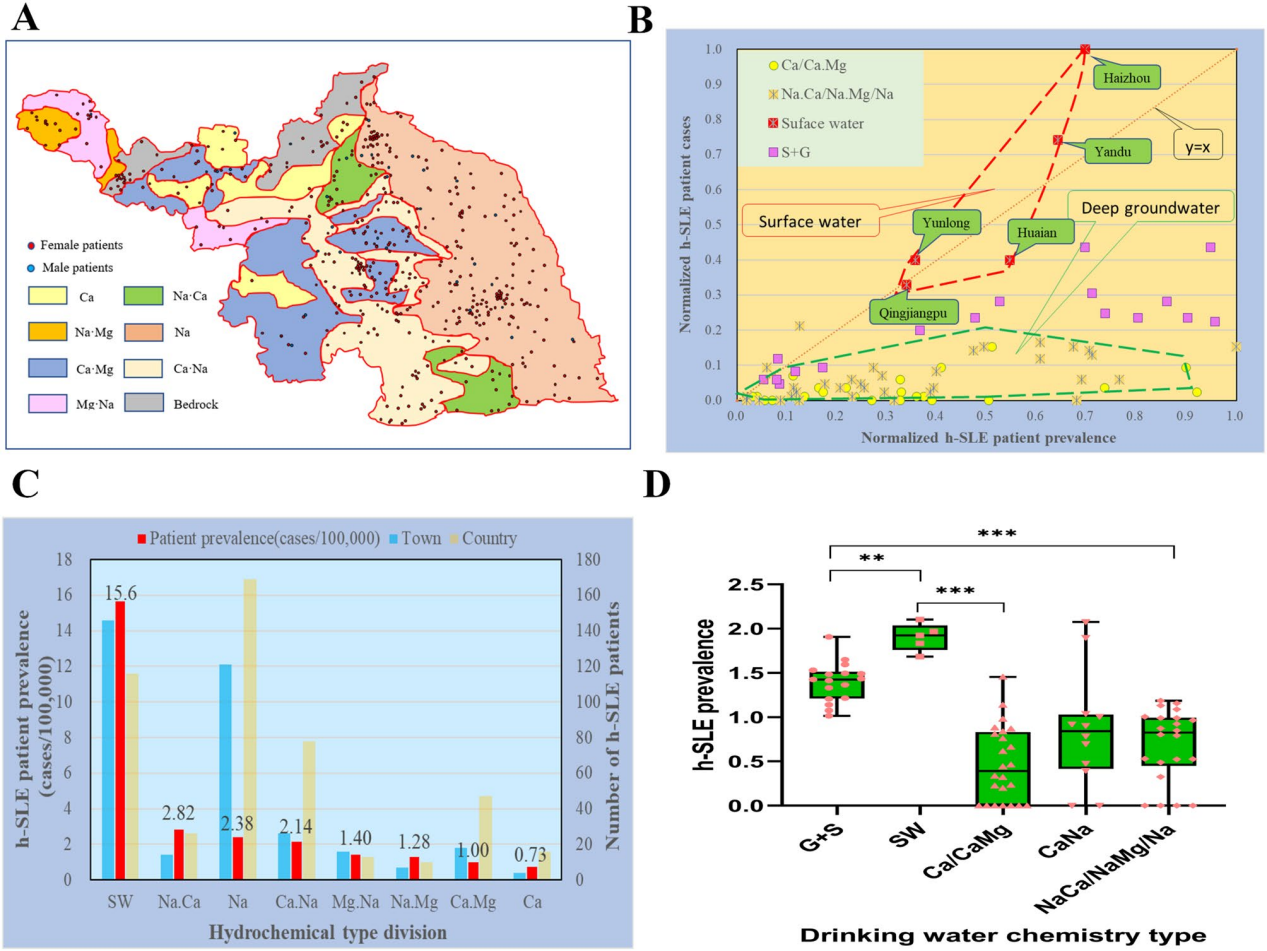
Relationship between the distribution of h-SLE patients and drinking water sources. (**A**) Distribution of groundwater chemistry types (2000s) and h-SLE patients in northern Jiangsu (prepared by WQ in Microsoft Excel 2019, referring to the cation distribution map of hydrochemical facies for the phreatic aquifer in the 2000s in the paper, https://doi.org/10.1007/s12665-015-4575-4). (**B**) Normalized relationship between the prevalence of h-SLE patients and the cases of h-SLE in different drinking water sources in northern Jiangsu. (**C**) Prevalence distribution of h-SLE patients with different chemical drinking water sources in Northern Jiangsu. (**D**) Significant difference analysis of the prevalence of h-SLE patients in water chemistry type. The prevalence of h-SLE patients conformed to the normal distribution after BOX-COX conversion (λ = 0.053) and Shapiro–Wilk Test (P value: 0.186 > 0.05). Statistical analysis was performed with one-way ANOVA. **P < 0.01, ***P < 0.001.

Generally, Ca-type and Ca·Mg-type groundwater are defined as unpolluted, and Na-type or high-Na-type groundwater is defined as polluted due to the high content of Na^+^ in surface water. To further analyze the relations between drinking water sources and the development of h-SLE, we defined different types of water pollution according to the distribution of cations in shallow groundwater in the north of Jiangsu in 2000 (Fig. 3A)^26^. To normalize the relationship between the patient prevalence and patient cases, we classified drinking water sources in the north of Jiangsu as: surface water, surface water & groundwater, and groundwater (Fig. 3B). Considering that groundwater may be polluted by surface water, Ca·Mg-type groundwater that is not polluted and Na-type groundwater that may be polluted were classified. The prevalence of h-SLE patients was highest in SW (surface water) area, reaching 15.6 cases per 100,000 people; while the prevalence of h-SLE patients was significantly lower in ground water area, of which the lowest was only 0.73 cases per 100,000 people in Ca-type area (Fig. 3C), and there was a significant difference in h-SLE patient prevalence among the five different types of drinking water, especially between SW and Ca-type and Ca·Mg-type (P < 0.001) (Fig. 3D). The number of cases in Na·Ca-type & Na·Mg-type groundwater area was between 2.82 and 1.28 per 100,000, which might be related to a certain degree of pollution to groundwater with the presence of Na. Taken together, these data supported that the development of SLE was probably triggered by the concentrated discharge of exhaust water.

### Correlations of pollution with clinical characteristics and mortality of h-SLE patients

**Figure 4:**
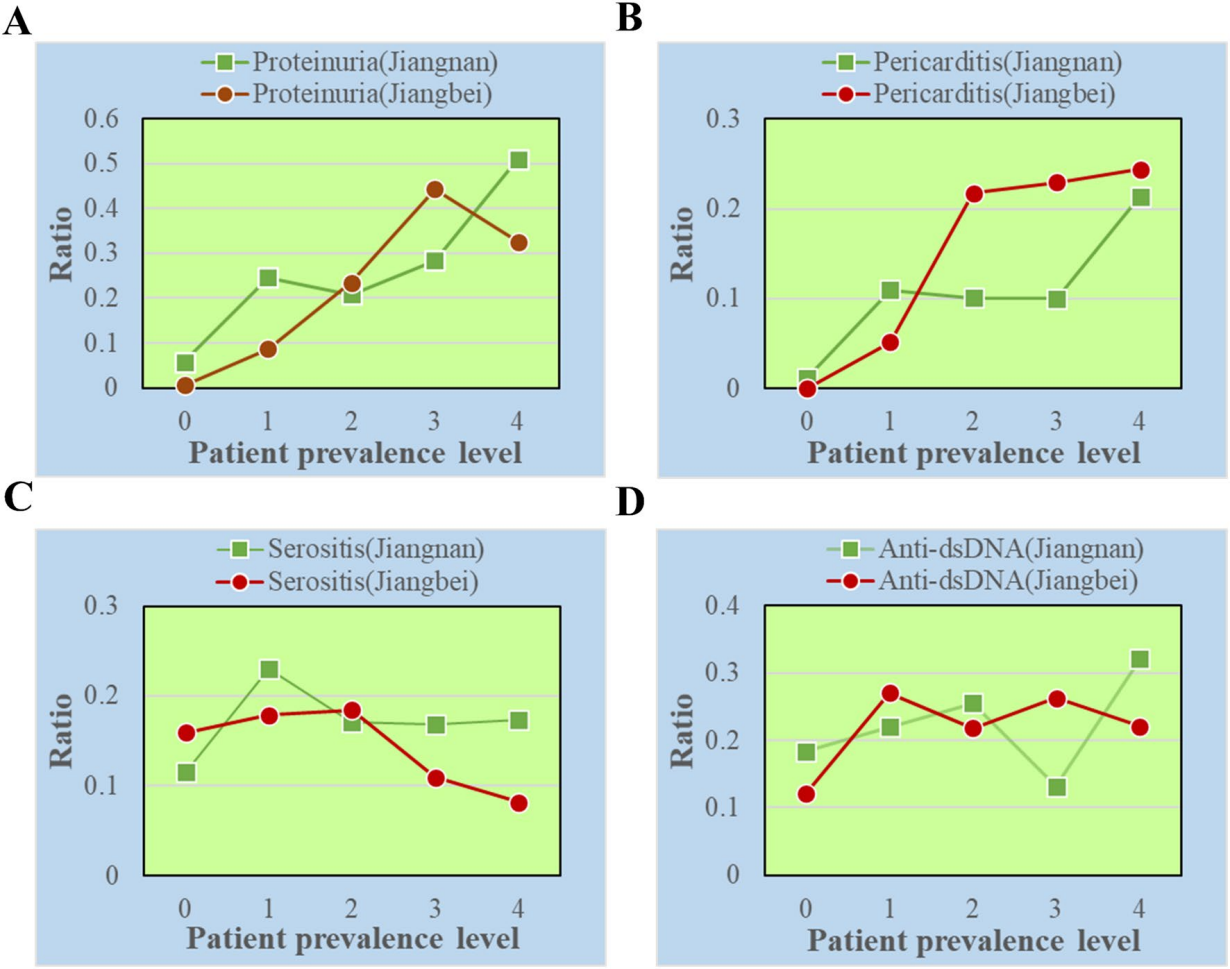
Proportion of clinical manifestation indicators of h-SLE patients with 5 prevalence levels in Jiangnan and Jiangbei. (**A**) Proteinuria. (**B**) Pericarditis. (**C**) Serositis. (**D**) Anti-dsDNA antibodies. The patient prevalence (cases per 100,000 people) was divided into 5 levels: level 0: prevalence < 2 (432 cases); level 1: 2 < prevalence < 3(443 cases); level 2: 3 < prevalence < 5(496 cases); level 3: 5 < prevalence < 10(503 cases); level 4: prevalence > 10(357 cases).

Jiangsu Province is distributed in the two regions of Jiangnan and Jiangbei by Yangtze River with obvious difference in climatic conditions and patient prevalence. We next determined the correlations between different h-SLE patient prevalence in Jiangnan and Jiangbei and their clinical characteristics (Fig. 4, Supplementary Fig. S2, Supplementary Tables S4 and S5). We first made a correlation analysis on clinical characteristics (Supplementary Fig. S3), and found the correlation coefficient between proteinuria and anti-dsDNA antibodies was 0.074 (p = 0.0005), however, the correlation coefficient of proteinuria with pericarditis (r = 0.03, p = 0.1596), fever (r = -0.0043, p = 0.8379), rash (r = 0.034, p = 0.1098); Anti-dsDNA with Arthritis (r = 0.0085, p = 0.6883), fever (r = 0.03, p = 0.1614), rash (r = -0.014, p = 0.5081) and dropsy (r = 0.025, p = 0.9068), respectively, indicating the presence of statistically independent variables. The ratio of patient with proteinuria and pericarditis significantly increased with the increase of patients' prevalence (Fig. 4A,B), indicating that environment pollution might play a role in the development of lupus nephritis, the most common cause of morbidity and mortality for lupus. Further, as the patient prevalence increases, the ratio of patient with anti-double stranded DNA (anti-dsDNA) auto-antibodies, a disease activity indicator, also increased (Fig. 4D), suggesting that environment pollution might affect the disease status of h-SLE patients. However, the overall trends were not consistent among the different manifestations, no significant difference was observed for clinical manifestations including polyserositis, arthritis, fever, rash, dropsy, and so on, this may be affected by differences in individual immunity in the region and the high degree of heterogeneity of SLE, in addition, gender, age, organ involvement, and differences in the sensitivity and specificity of the biomarkers themselves also affected the trend of clinical manifestations^27–31^. Considering that NOx is the only common substance both in air and water pollution, and regardless of air or drinking water, the area with high NOx concentration has a high percentage of patients with pericarditis and proteinuria, our data gave us a hint that NOx in air and water pollution may be one of the main environmental triggers of SLE.

To investigate the role of environmental pollution in mortality rate of h-SLE patients, we followed up h-SLE patients mainly in 2010 and 2015, the number of interviews was 1251 cases (survivors:1043, death:208), and the number of missing interviews in the survey was 980, accounting for 44% of the total number of patients. The death rate (24.7%) of level 4 (prevalence > 10 cases/100,000 people) was significantly higher than that of other levels (15.3% & 17.5% 14.5% & 14.2%, Supplementary Table S6), distributed in Runzhou (prevalence: 26.6), Jingkou (prevalence: 20.0), Binhu (prevalence: 13.0), Haizhou (prevalence:10.9) and Dantu (prevalence: 10.1). Among them, Runzhou, Jingkou and Dantu all belong to Zhenjiang City, a place for the concentrated emission of chemical plants, and Binhu, Wuxi, where the source of drinking water is eutrophic Tai Lake, had not only a high prevalence, but also a high mortality rate. Together, our data showed that the mortality rate was positively correlated with the prevalence of h-SLE patients, and pollution not only induced a high h-SLE patient prevalence but also a higher mortality rate.

## Discussion

Our data demonstrated that h-SLE patient prevalence was highest in districts with severe air or drinking water pollution. The proportion of patients with pericarditis and proteinuria was positively correlated with the h-SLE patient prevalence, which might be attributed to the concentrated NOx contamination in the air and drinking water. It existed regional difference in the h-SLE patient prevalence, with the ratio of highest to lowest prevalence reaching 240. h-SLE high-prevalence districts also had high mortality rates and the prevalence of lupus nephritis, indicating that the prevalence and characteristics of SLE were related to the living environment, especially the concentrated discharge of air pollution and substandard drinking water.

Here we demonstrated that 6 out of 15 districts with high h-SLE patient prevalence were related to air pollution full of PM10, PM2.5, SO_2_, NOx, CO, O_3_, etc. In addition, the h-SLE prevalence was also related to the polluted drinking water sources, for example, drinking water source of four high-prevalence districts in Wuxi City is Tai Lake, in which excessive nitrogen and phosphorus cause serious eutrophication of the water body. The pollutants in drinking water are mainly nitrate nitrogen, ammonia nitrogen, potassium permanganate, phosphorus, COD, chloride, sulfide, etc.^32^, but the common feature is that the nitrate nitrogen exceeds the standard. NOx is a substance that coexists in air pollution and drinking water source pollution, indicating that whether it is air pollution or water pollution, NOx should be the culprit that induces the prevalence of SLE.

The mechanisms by which NOx participates in the pathogenesis of SLE still largely unknown. Carbon monoxide (NO) in the human body is derived from L-arginine and catalyzed by nitric oxide synthase^33^. The subtype of nitric oxide synthase-2 may be affected by oxidative stress and cytokines produced after inhalation of air pollutants^34^, and the acute effect of PM2.5 exposure on FeNO in c-SLE patients suggests that these patients have a higher degree of epithelial-mediated airway inflammation after exposure to fine particles^35,36^. Increasing evidence suggests that vascular effects such as estrogen increasing uterine arterial blood flow may be mediated by NO^37^. A multivariate analysis in Taiwan showed that NO_2_ exposure is positively correlated with the incidence of SLE, and this may be caused by chronic exposure to NO_2_, which may cause inflammation and increase the level of inflammatory factors such as interleukin-6 (IL-6)^38^. In vitro experiments showed that exposure under O_3_ and NO_2_ in the cultured nasal mucosa increase the production of pro-inflammatory cytokines tumor necrosis factor (TNF-α) and IL-6 by inducing an inflammatory cascade^39,40^, and TNF-α stimulates the production of other inflammatory molecules including IL-6, IL-6 in turn induces the production of IL-6 producing acute-phase proteins, including CRP and fibrinogen, thereby participating in autoimmune inflammation^41,42^. Studies have shown that as an active oxidant, peroxynitrite produced by nitric oxide (NO) and superoxide anion (O_2_^**·**–^) is oxidized by proteins and DNA damage (including DNA strand breaks and base modifications) effective triggers can activate the ribozyme poly ADP ribose polymerase (PARP), leading to energy expenditure and apoptosis/necrosis^43,44^. In chronic inflammatory diseases, the peroxynitrite formed by phagocytes may cause damage to DNA, generates new epitopes, leads to the production of autoantibodies, and participates in the development of autoimmune diseases such as SLE^45^. In addition, NOx stimulation leads to changes in biochemical epigenetic modifications, which may be a potential mechanism for the onset of SLE^46^, and NOx-mediated oxidative stress has been shown to inhibit T cell ERK pathway signaling, leading to DNA demethylation, immune gene upregulation, and self-response, and may cause SLE in genetically susceptible individuals^47^.

Previous studies have suggested that economic development, economic income, and mental stress could be related to the progression of SLE^48,49^. In order to study the relationship between the h-SLE patient prevalence distribution and the economic development, we conducted a correlation analysis between the total night light observed with night light remote sensing in 2003 and the prevalence of h-SLE patients, night light remote sensing is highly related to economic development, the higher the brightness, the higher the degree of economic development, and the brightness can accurately reflect the economic development of the region and the average living standard of the people. Our data showed that there was no obvious correlation between the prevalence of h-SLE patients and the total night light (Supplementary Fig. S4A) and there was no significant difference in h-SLE patient prevalence among the five groups of Night light value (P > 0.05) (Supplementary Fig. S4B), for example, in Kunshan, Suzhou, where the total night light was the highest, the prevalence of h-SLE patients was very low. The normalized relationship between the amount of night light and h-SLE patient prevalence showed that there was no relationship between them (Supplementary Fig. S4C, R^2^ = 7 × 10^–5^). The analysis of the weighted value of night light in 16 districts showed that the night light value was not related to the prevalence of h-SLE patients (Supplementary Fig. S4D). To further confirm the role of economic level and living standard on the prevalence and mortality of h-SLE patients in 96 districts of Jiangsu province, we performed a statistical analysis of the effect of this inclusive difference between rural and urban areas on the prevalence of h-SLE (Supplementary Table S7 and Fig. S5), and found no significant difference in h-SLE patient prevalence between urban and rural areas except for the age (35.99 ± 12.79 & 34.41 ± 12.34, p = 0.0063) (Supplementary Table S7), which was consistent with the finding that there was no significant difference between night light values and h-SLE prevalence. In addition, there was no significant correlation between mortality and night light value and no significant difference in mortality between the groups divided by the night light values (Supplementary Fig. S4E,F). In conclusion, our data suggested that economic level and people's living standard were not the main causes of prevalence and mortality of h-SLE patients in the study area of Jiangsu Province. In our study, Jiangsu region rather than national or even worldwide patient data, hospitalized patients rather than all lupus patients, intercept selection bias of luminous data, and not yet reaching the threshold of triggering the disease of the economic level in Jiangsu province might contribute to the negative data between the prevalence and mortality of h-SLE patients and night light value.

In general, our data evaluated the relations between exposure under air and drinking water pollution and the h-SLE patient prevalence and mortality. We provided evidence that NOx in polluted air and drinking water may be one of the important predispositions of h-SLE, and pollution not only induced a high h-SLE patient prevalence but also a higher mortality rate, especially for patients with renal involvement. Limitations of the study are as followings: Firstly, our longitudinal database only enrolled hospitalized SLE patients in Jiangsu Province, lacking the outpatient samples, differences in the clinical manifestations of the two group patients (hospitalized patients & outpatients), including gender, age, organ involvement, disease activity and medications, might affect the data analysis. Secondly, some patients were diagnosed for the first time while some patients were in the course of the disease, and some inclusion biases might cause deviations in the results. Further limitations stem from a causality link, albeit biological plausible, cannot be proven yet due to lack of control group. Finally, the paper is more of a cross-sectional study from the perspective of geospatial and further longitudinal research is needed to confirm these findings and evaluate potential biological mechanisms.

## Supplementary Information

Supplementary Information.

## Data Availability

The datasets used and analyzed during the current study are available from the corresponding author upon reasonable request.
