# Supplementary material for: The relationship of polluted air and drinking water sources with the prevalence of systemic lupus erythematosus: a provincial population-based study
Source: Sci Rep. 2021 Sep 20;11:18591. doi: 10.1038/s41598-021-98111-8 (PMC8452734; doi:10.1038/s41598-021-98111-8)
Supplement: Supplementary file 1 — Supplementary Information. [file 41598_2021_98111_MOESM1_ESM.docx]

**Title:** The relationship of polluted air and drinking water sources with the prevalence of systemic lupus erythematosus: a provincial population-based study

**Authors:** Jiaqi Chen, Wenqiang Qu, Li Sun, Jiansheng Chen, Wei Kong, Fan Wang, Wenyou Pan, Lin Liu, Min Wu, Fuwan Ding, Huaixia Hu, Xiang Ding, Hua Wei, Yaohong Zou, Xian Qian, Meimei Wang, Jian Wu, Juan Tao, Jun Tan, Zhanyun Da, Miaojia Zhang, Jing Li, Jun Liang, Xuebing Feng, Linyu Geng & Lingyun Sun

**Supplementary Figures**


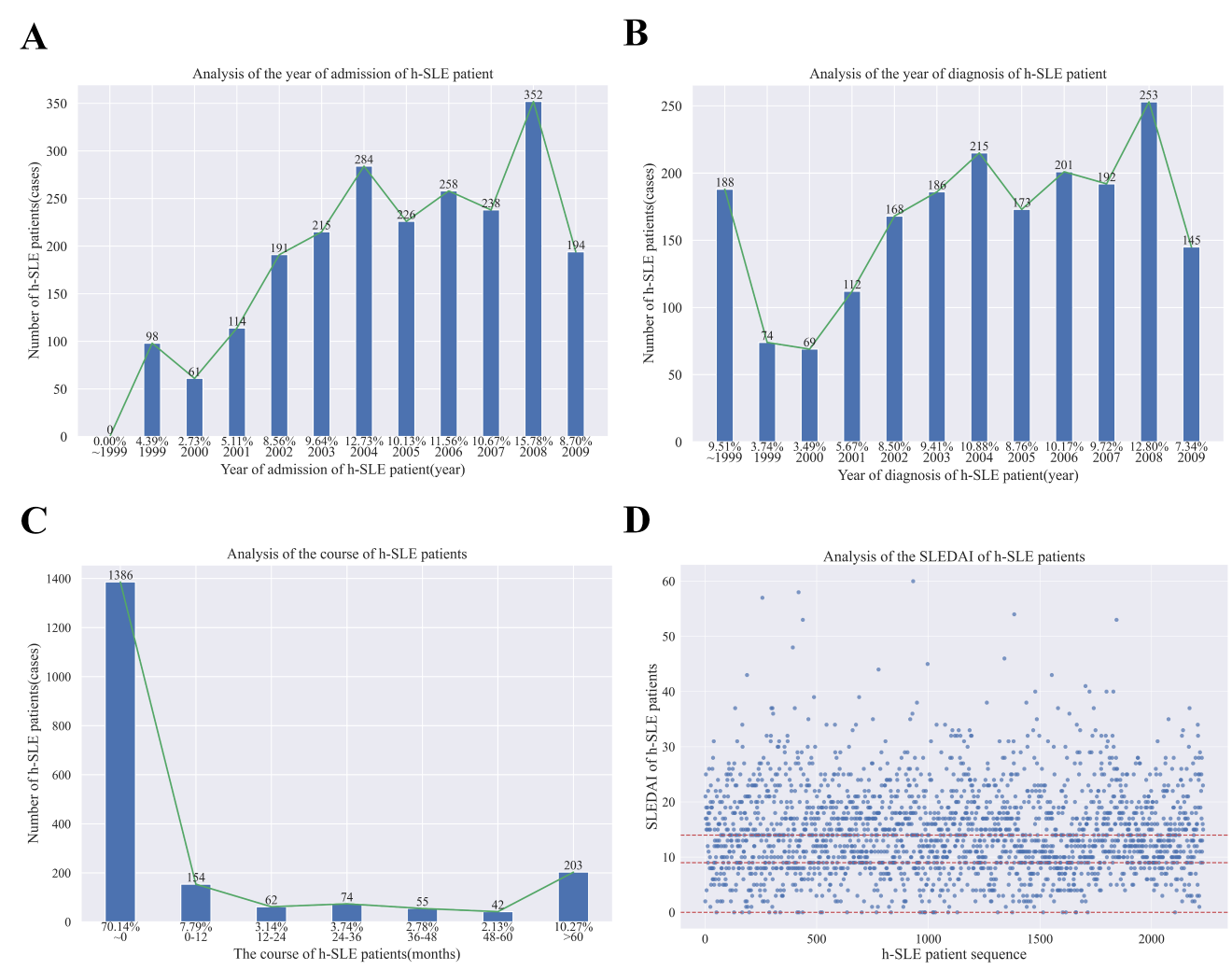


**Figure 1.** **Basic information of h-SLE patients. (A)** Year of admission. **(B)** Year of diagnosis. **(C)** Disease course. **(D)** SLEDAI.


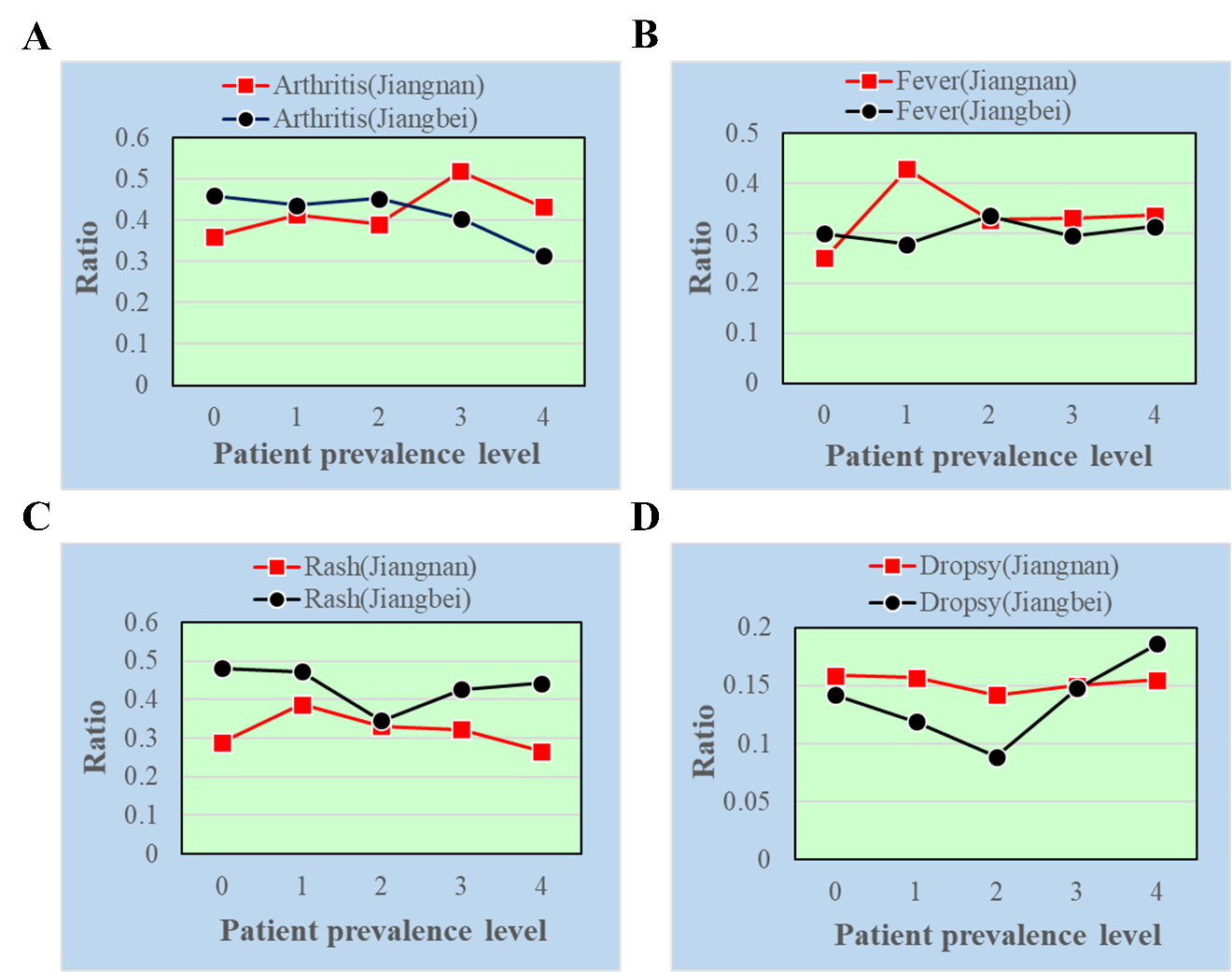


**Figure 2. Proportion of clinical manifestations of h-SLE patients with 5 prevalence levels in Jiangnan and Jiangbei.**  (**A**) Arthritis. (**B**) Fever. (**C**) Rash. (**D**) Dropsy. The patient prevalence was divided into 5 levels: level 0: prevalence<2 (432 cases); level 1: 2<prevalence<3(443 cases); level 2:3<prevalence<5(496 cases); level 3: 5<prevalence<10(503 cases); level 4: prevalence >10(357 cases).

**
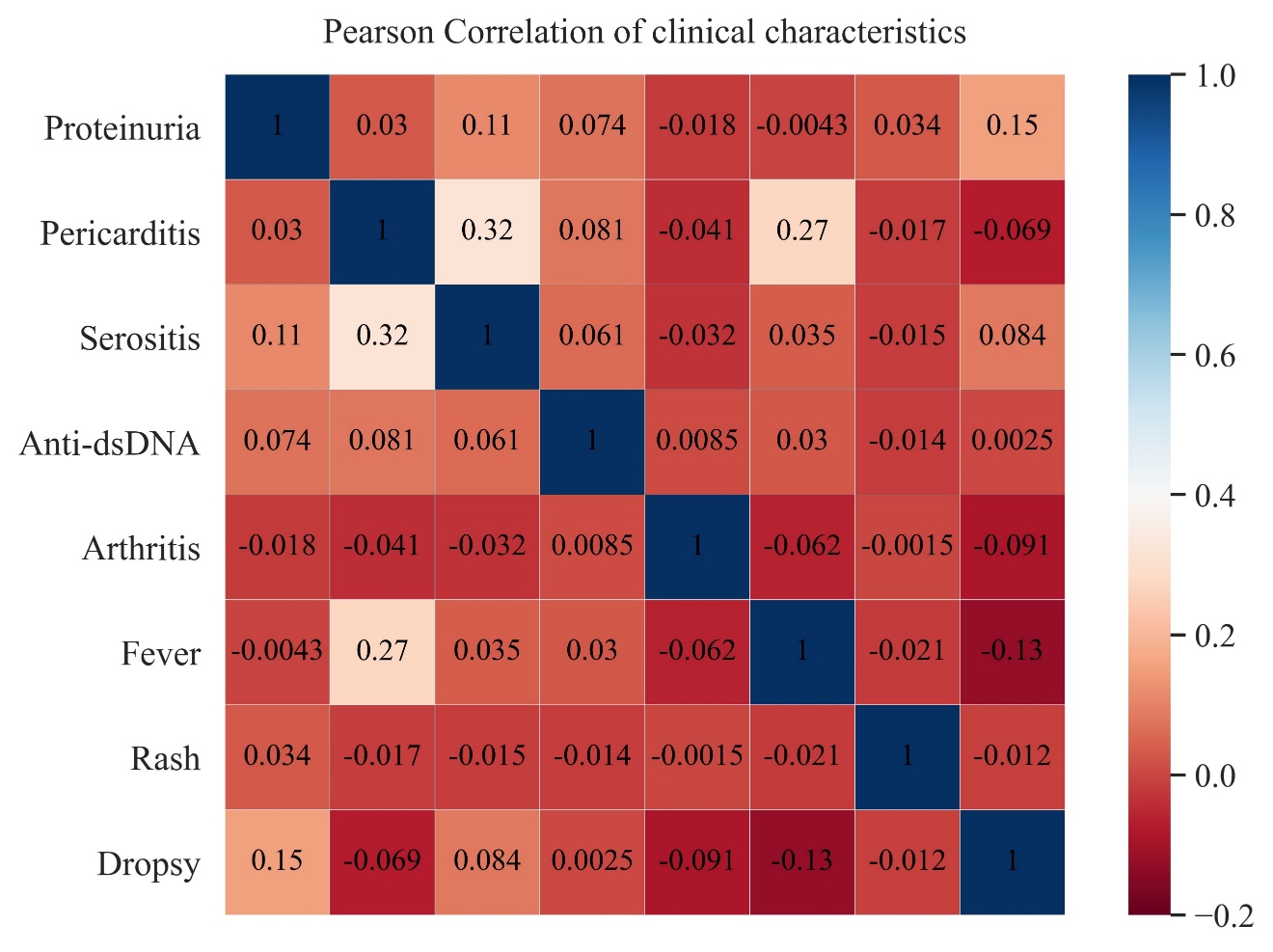
**

**Figures 3. Correlation coefficient analysis of clinical characteristics**

**
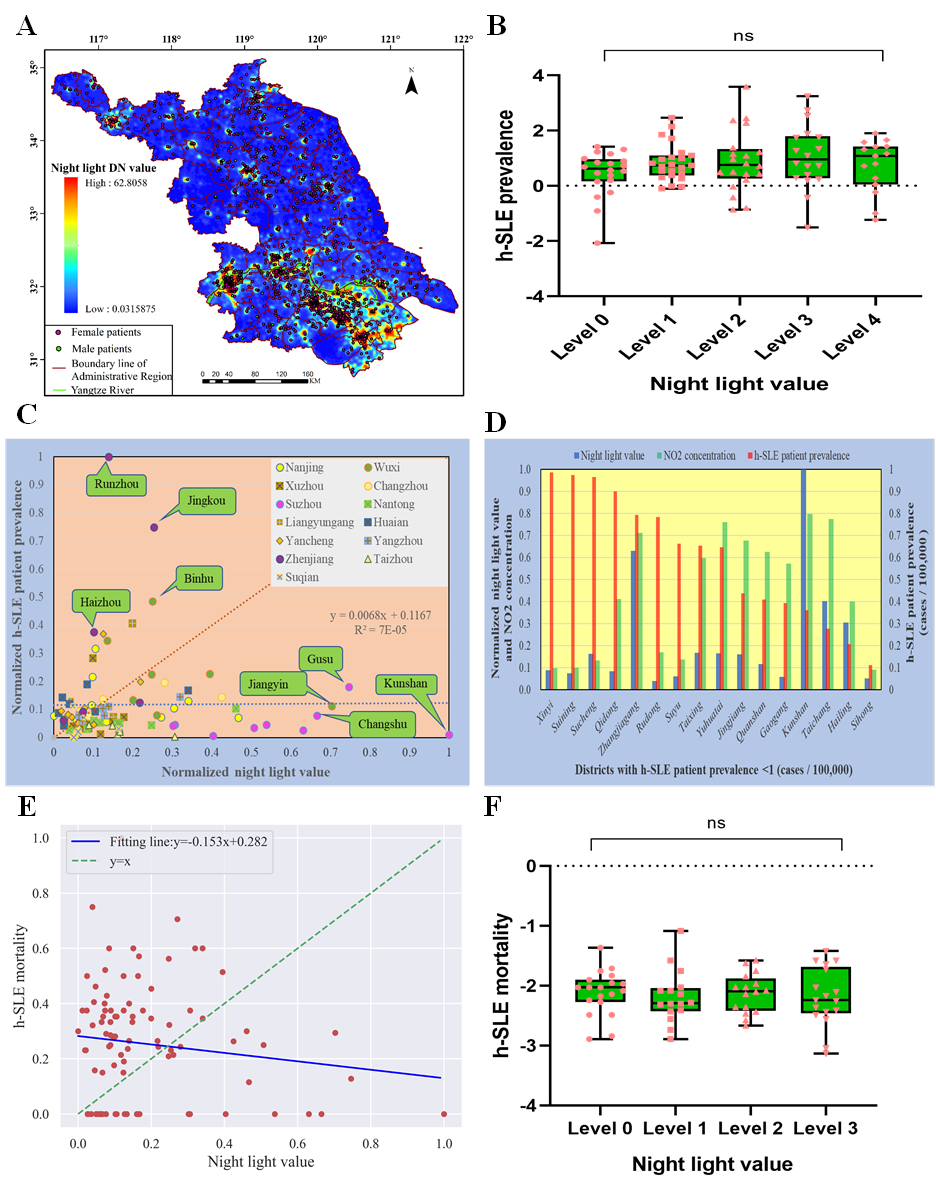
**

**Figure 4. The relationship between the prevalence and mortality of h-SLE patients and the total night light.** (**A**) Distribution of h-SLE patients in Jiangsu Province during 1999-2009 and night light values in 2003 (prepared by WQ in ArcMap 10.2, https://www.esri.com/zh-cn/arcgis/products/arcgis-pro/resources). **(B)** Significant difference analysis of the prevalence of h-SLE patients in night light value group. (**C**) Normalized relationship between h-SLE patient prevalence and night light values. (**D**) The distribution of night light, NO_2_ concentration and population density in administrative districts with h-SLE patient prevalence <1. (E) Normalized relationship between h-SLE patient mortality and night light values. (F) Significant difference analysis of the mortality of h-SLE patients in night light value group. Note: The prevalence and mortality of h-SLE conforms to the normal distribution after BOX-COX conversion (λ=0.053 & 0.024) and Shapiro-Wilk Test (P value:0.186>0.05 & 0.913>0.05). Statistical analysis was performed with one-way ANOVA. ns, not significant.


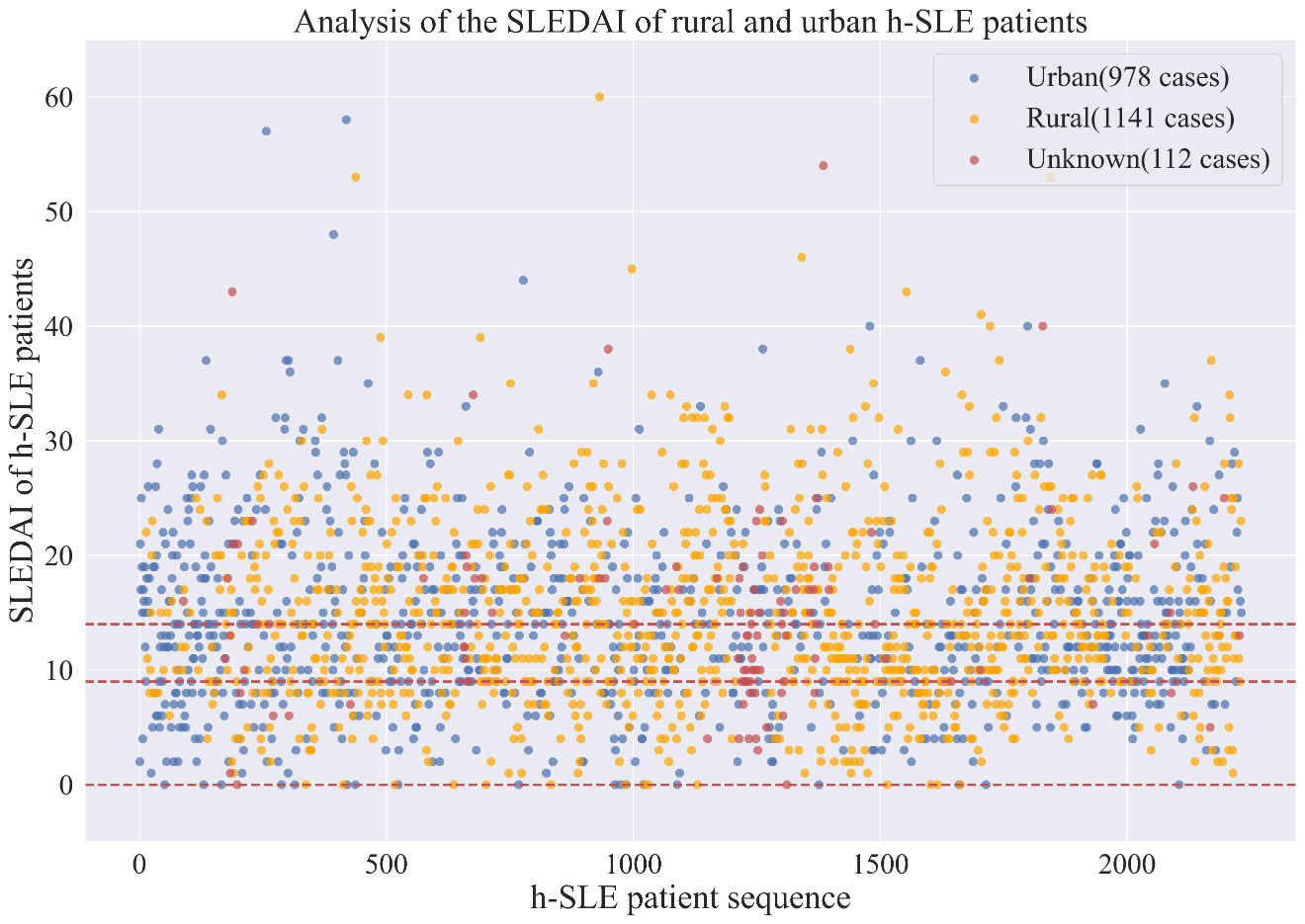


**Figure 5.** **SLEDAI distribution of h-SLE patients in rural and urban areas.**

**Supplementary Tables**

**Table 1.** **Statistical analysis of SLEDAI classification of h-SLE patients**

| **SLEDAI** | **Number** |
| --- | --- |
| **0-9 (Mild)** | 606（27.2%） |
| **10-14 (Moderate)** | 625（28.0%） |
| **15- (Severe)** | 1000（44.8%） |

SLEDAI, SLE disease activity index.

**Table 2. The demographic and clinical characteristics of h-SLE patients with different courses**

| **Variable** | **Total**  **(n=1976)** | **First**  **(n=1386)** | **Along**  **(n=590)** | **P value** |
| --- | --- | --- | --- | --- |
| **Age, years (Mean±SD)** | 35.00±12.61 | 34.82±12.53 | 35.41±12.79 | 0.4141 |
| **Female (%)** | 1824(92.31) | 1275(91.99) | 549(93.05) | 0.4376 |
| **Male (%)** | 152(7.69) | 111(8.01) | 41(6.95) | 0.4736 |
| **SLEDAI on admission** | 14.67±7.85 | 15.12±7.72 | 13.61±8.06 | **0.0000** |
| **Organ involvements** |  |  |  |  |
| Mucocutaneous | 1296(65.59) | 912(65.80) | 384(65.08) | 0.7988 |
| Neuropsychiatric | 125(6.33) | 82(5.92) | 43(7.29) | 0.2958 |
| Musculoskeletal | 1109(56.12) | 811(58.51) | 298(50.51) | **0.0012** |
| Cardiopulmonary | 420(21.26) | 314(22.66) | 106(17.97) | **0.0231** |
| Gatrointestinal | 98(4.96) | 67(4.83) | 31(5.25) | 0.7791 |
| Ocular | 9(0.46) | 7(0.51) | 2(0.34) | 0.8913 |
| Renal | 1015(51.37) | 661(47.69) | 354(60.00) | **0.0000** |
| Haematological | 956(48.38) | 720(51.95) | 236(40.00) | **0.0000** |

SLE, Systemic lupus erythematosus; SLEDAI, SLE disease activity index. Statistical analysis was performed with the Mann–Whitney U-test and the χ2 test.

**Table 3. The relevant parameters in districts with h-SLE patient prevalence > 5 (cases per 100,000 people)**

| **Districts** | **patients** | **Population density** | **Patient**  **prevalence** | **NO_2_ concentration** | **Night light value** | **Drinking water source** | **Air pollution source** |
| --- | --- | --- | --- | --- | --- | --- | --- |
| Runzhou, Zhenjiang | 68 | 2867 | 26.65 | 1075 | 7726 | Yangtze River  Ⅰ~Ⅲ | Factory  Motor vehicle |
| Jingkou, Zhenjiang | 78 | 3122 | 19.99 | 950 | 13289 | Yangtze River  Ⅰ~Ⅲ | Factory  Motor vehicle |
| Binhu,  Wuxi | 93 | 1140 | 12.99 | 835 | 13073 | Tai Lake  Ⅲ^+^~Ⅴ^+^ | Motor vehicle |
| Haizhou, Lianyungang | 86 | 1129 | 10.87 | 413 | 10677 | Rose River  Ⅲ^+^~Ⅴ^+^ | Motor vehicle |
| Dantu, Zhenjiang | 32 | 513 | 10.11 | 898 | 5884 | Yangtze River  Ⅰ~Ⅲ | Factory  Motor vehicle |
| Yandu, Yancheng | 63 | 627 | 9.90 | 382 | 7032 | Boa River  Ⅲ^+^~Ⅴ^+^ | Motor vehicle |
| Liangxi,  Wuxi | 89 | 13448 | 9.26 | 940 | 7547 | Tai Lake  Ⅲ^+^~Ⅴ^+^ | Factory  Motor vehicle |
| Xuanwu, Nanjing | 51 | 7922 | 8.53 | 1011 | 6071 | Yangtze River  Ⅰ~Ⅲ | Factory  Motor vehicle |
| Yunlong, Xuzhou | 34 | 3730 | 7.61 | 636 | 5741 | Xiaoyanhe  Ⅲ^+^ | Factory  Motor vehicle |
| Xinwu,  Wuxi | 35 | 2587 | 6.15 | 909 | 20167 | Tai Lake  Ⅲ^+^~Ⅴ^+^ | Factory  Motor vehicle |
| Xishan,  Wuxi | 43 | 1773 | 6.08 | 871 | 12993 | Tai Lake  Ⅲ^+^~Ⅴ^+^ | Factory  Motor vehicle |
| Gulou,  Nanjing | 65 | 20936 | 5.86 | 1069 | 5729 | Yangtze River  Ⅰ~Ⅲ | Factory  Motor vehicle |
| Tinghu, Yancheng | 37 | 858 | 5.39 | 336 | 11619 | Lake water  Ⅲ^+^~Ⅴ^+^ | Motor vehicle |
| Xinbei, Changzhou | 37 | 1363 | 5.34 | 827 | 14550 | Yangtze River  Ⅰ~Ⅲ | Factory  Motor vehicle |
| Huaian,  Huaian | 49 | 654 | 5.16 | 377 | 5078 | Hongze Lake  Ⅲ^+^~Ⅴ^+^ | Motor vehicle |

Patients, cases; Population density, person/km^2^; Patient prevalence, cases per 100,000 people; Since the Night light value and NO_2_ concentration data have been processed, there is no clear unit, and the value indicates the level of brightness and concentration.

**Table 4. Clinical characteristics of patients with different prevalence in Jiangnan**

| **Patient**  **prevalence** | **Number of patients** | **Pericarditis**  **(%)** | **Anti-ds-DNA (%)** | **Serositis (%)** | **Proteinuria (%)** | **Arthritis**  **(%)** | **Fever**  **(%)** | **Rash**  **(%)** | **Dropsy**  **(%)** |
| --- | --- | --- | --- | --- | --- | --- | --- | --- | --- |
| **＜2** | 87 | 1.1 | 18.4 | 11.5 | 5.7 | 36.1 | 25.1 | 29.0 | 15.9 |
| **2-3** | 191 | 11.0 | 22.0 | 23.0 | 24.6 | 41.4 | 42.9 | 38.7 | 15.7 |
| **3-5** | 317 | 10.1 | 25.6 | 17.0 | 20.8 | 39.1 | 32.8 | 33.1 | 14.2 |
| **5-10** | 320 | 10.0 | 13.1 | 16.9 | 28.4 | 51.9 | 33.1 | 32.2 | 15.0 |
| **＞10** | 271 | 21.4 | 32.1 | 17.3 | 50.9 | 43.2 | 33.6 | 26.6 | 15.5 |

Patient prevalence, Cases per 100,000 people.

**Table 5. Clinical characteristics of patients with different** **prevalence in Jiangbei**

| **Patient prevalence** | **Number of patients** | **Pericarditis**  **(%)** | **Anti-ds-DNA (%)** | **Serositis (%)** | **Proteinuria (%)** | **Arthritis**  **(%)** | **Fever**  **(%)** | **Rash**  **(%)** | **Dropsy**  **(%)** |
| --- | --- | --- | --- | --- | --- | --- | --- | --- | --- |
| **＜2** | 345 | 0.0 | 12.2 | 15.9 | 0.6 | 46.0 | 30.0 | 48.1 | 14.2 |
| **2-3** | 252 | 5.2 | 27.0 | 17.9 | 8.7 | 43.7 | 27.8 | 47.2 | 11.9 |
| **3-5** | 179 | 21.8 | 21.8 | 18.4 | 23.5 | 45.3 | 33.5 | 34.6 | 8.9 |
| **5-10** | 183 | 23.0 | 26.2 | 10.9 | 44.3 | 40.4 | 29.5 | 42.6 | 14.8 |
| **＞10** | 86 | 24.4 | 22.1 | 8.1 | 32.6 | 31.4 | 31.4 | 44.2 | 18.6 |

Patient prevalence, Cases per 100,000 people.

**Table 6. Survival statistics of h-SLE patients in Jiangsu Province from 1999 to 2009 in 2015**

| **Patient prevalence** | **Number of**  **patients** | **Number of visitors** | **Number of deaths** | **Number of survivors** | **mortality rate（%）** | **Visit rate（%）** |
| --- | --- | --- | --- | --- | --- | --- |
| **＜2** | 432 | 239 | 34 | 205 | 14.2 | 55.3 |
| **2-3** | 443 | 256 | 37 | 219 | 14.5 | 57.8 |
| **3-5** | 496 | 314 | 55 | 259 | 17.5 | 63.3 |
| **5-10** | 503 | 288 | 44 | 244 | 15.3 | 57.3 |
| **＞10** | 357 | 154 | 38 | 116 | 24.7 | 43.1 |

Patient prevalence, Cases per 100,000 people.

**Table 7. The demographic and clinical characteristics of h-SLE patients in rural and urban areas**

| **Variable** | **Total**  **(n=2119)** | **Urban**  **(n=978)** | **Rural**  **(n=1141)** | **P value** |
| --- | --- | --- | --- | --- |
| **Age, years (Mean±SD)** | 35.14±12.57 | 35.99±12.79 | 34.41±12.34 | **0.0063** |
| **Female (%)** | 1957(92.35) | 909(92.10) | 1048(91.85) | 0.3875 |
| **Male (%)** | 162(7.65) | 69(6.99) | 93(8.15) | 0.3875 |
| **SLEDAI on admission** | 14.71±8.36 | 14.36±7.81 | 14.80±7.97 | 0.2850 |
| **Organ involvements** |  |  |  |  |
| Mucocutaneous | 1375(64.89) | 613(62.11) | 762(66.78) | 0.0539 |
| Neuropsychiatric | 131(6.18) | 56(5.67) | 75(6.75) | 0.4735 |
| Musculoskeletal | 1183(55.83) | 538(54.51) | 645(56.53) | 0.5105 |
| Cardiopulmonary | 456(21.52) | 206(20.87) | 250(22.91) | 0.6744 |
| Gatrointestinal | 105(4.96) | 47(4.76) | 58(5.08) | 0.8469 |
| Ocular | 9(0.42) | 5(0.51) | 4(0.35) | 0.8166 |
| Renal | 1093(51.58) | 490(49.65) | 603(52.85) | 0.2235 |
| Haematological | 1002(47.29) | 453(45.90) | 549(48.12) | 0.4341 |

SLEDAI, SLE disease activity index. Statistical analysis was performed with the Mann–Whitney U-test and the χ2 test.
